# Antimicrobial Resistance and Phylogenetic Analysis of Multidrug-Resistant Non-Typhoidal *Salmonella* Isolates from Different Sources in Southern Vietnam

**DOI:** 10.3390/antibiotics14050489

**Published:** 2025-05-10

**Authors:** Daria Starkova, Svetlana Egorova, Ludmila Suzhaeva, Truong Quang Nguyen, Lidiia Kaftyreva, Maria Makarova, Samida Zhamborova, Dmitrii Polev, Alina Saitova, Vu Hoang Nguyen, Tram Khac Vo, Long Thanh Nguyen

**Affiliations:** 1 Laboratory of Identification of the Pathogens, St. Petersburg Pasteur Institute, 197101 St. Petersburg, Russia; egorova@pasteurorg.ru (S.E.); suzhaeva@pasteurorg.ru (L.S.); 2 Pasteur Institute of Ho Chi Minh City, Ho Chi Minh City 70000, Vietnam; truongnq@pasteurhcm.edu.vn (T.Q.N.); vunh@pasteurhcm.edu.vn (V.H.N.); 3 Laboratory of Intestinal Infections, St. Petersburg Pasteur Institute, 197101 St. Petersburg, Russia; kaftyreva@pasteurorg.ru (L.K.); makarova@pasteurorg.ru (M.M.); zhamborova@pasteurorg.ru (S.Z.); 4 Laboratory of Metagenomics Research, St. Petersburg Pasteur Institute, 197101 St. Petersburg, Russiasaitova@pasteurorg.ru (A.S.); 5 Department of Animal Husbandry and Veterinary Medicine of Ho Chi Minh City, Ho Chi Minh City 743000, Vietnam; vktram@chicuccntyhcm.gov.vn (T.K.V.); ntlong@chicuccntyhcm.gov.vn (L.T.N.)

**Keywords:** non-typhoidal *Salmonella*, Vietnam, livestock, antimicrobial resistance genes, whole-genome sequencing, multidrug-resistant *Salmonella*

## Abstract

**Background/Objectives:** Non-typhoidal *Salmonella* (NTS) is one of the most common causative agents of food poisoning in Vietnam, and contaminated livestock meat poses a major risk to human health. The present study aims to provide the genetic characteristics of NTS with a particular focus on antimicrobial resistance and determine phylogenetic relationships between isolates from different sources in Southern Vietnam based on whole-genome sequencing (WGS) data. **Methods:** A total of 49 NTS isolates from pork/broiler meat, pigs, chickens, and humans were collected in Ho Chi Minh City and four provinces of Southern Vietnam. Phenotypic antimicrobial susceptibility testing (AST) and WGS for all isolates were performed. **Results**: As a result, 14 different serotypes were identified, among which *S.* Typhimurium and its monophasic variant were the dominant serotypes for human and pig sources. All chicken samples belonged to *S*. Indiana, whereas *S*. Infantis predominated in broiler meat. AST results revealed that 98% of isolates were multidrug resistant. NTS strains isolated from poultry and pigs exhibited resistance to the highest priority antimicrobials—quinolones and polymyxin, as well as to β-lactams, aminoglycosides, tetracycline, and sulfonamide, which are considered to be critical for the treatment of severe diseases. **Conclusions:** The results highlight the utmost importance of issues related to the selection, spreading, and transmission of multi-resistant strains from animals to humans.

## 1. Introduction

Non-typhoidal *Salmonella* (NTS) is a food-borne pathogen that causes a significant health and economic global burden worldwide, accounting for 4.38 million DALYs (Disability-Adjusted Life Years) (2007–2015) [1,2,3]. Being zoonotic, NTS is hosted by livestock and, due to further contamination of the environment, water, and food, manifests as a leading causative agent in food poisoning worldwide [1,2,4]. Among different food sources, contaminated poultry and pork products pose a main risk to human health as they participate in different food chain stages [2,3].

The use of antibiotics in the food animal sector plays a key role in the emergence of multidrug-resistant (MDR) bacteria: so far, approximately 80% of farm animals are treated with broad-spectrum antibiotics, which, given the increasing globalization of the livestock industry, exerts strong selective pressure on bacterial populations. Through adaptive evolution, *Salmonella* have developed diverse and complex resistance mechanisms that facilitate the rapid acquisition and spread of antibiotic resistance across diverse host species. Horizontal gene transfer, point mutations, the presence of heavy metal resistance genes and virulence genes, compensatory mechanisms, epistatic interactions, formation of biofilms, and the activation of efflux pumps, – all contribute to the MDR evolution in *Salmonella*. Moreover, it is suggested that *Salmonella* can exchange resistance genes with other bacteria of the Enterobacteriaceae family during passage through the intestine [3,4,5].

The exposure of human populations to antibiotic-resistant bacteria is facilitated by the consumption of animal products or direct contact with contaminated sources, both of which resulted from inadequate hygiene practices throughout the food chain production. This situation gives rise to global human health issues, including treatment failure, limited antimicrobial choices, increased morbidity, heightened disease burdens, outbreaks, and a higher risk of mortality.

As a global health problem, key international documents and guidelines were developed to prevent and reduce the use of antibiotics in animal agriculture: the World Health Organization (WHO) Guidelines on the Use of Medically Important Antimicrobials in Food-Producing Animals presented recommendations on the use of medically important antimicrobials in food-producing animals to preserve their effectiveness in human and veterinary medicine; the Food and Agriculture Organization (FAO) and the World Organization for Animal Health (OIE) provide guidelines for monitoring the use of antimicrobials across the human, animal and plant sectors; the Global Repository of Available Guidelines for Prudent Use of Antimicrobials compiled by the World Veterinary Association created a global repository of available guidelines for responsible use of antimicrobials in animal health; the ASEAN Guidelines for Prudent Use of Antimicrobials in Livestock are intended for consideration by ASEAN Member States for developing national strategies to combat antimicrobial resistance through the prudent use of antimicrobials in livestock sectors; and the 79th United Nations General Assembly Declaration committing to a significant reduction in the global quantity of antimicrobials use in the agrifood system by 2030 [4,5,6,7,8].

Although the prevalence of multidrug-resistant *Salmonella* varies geographically, the incidence of MDR NTS worldwide is rising steadily. The situation is further exacerbated by growing resistance to critical classes of antibiotics that are essential for severe infection treatment [7]. Globally, the highest MDR rates have been reported in South and Southeast Asia (Thailand—69%, Taiwan—53.8%, India—68%, Bangladesh—94%) and sub-Saharan Africa (77.2–87.4%), which are considered super regions for the global burden of multidrug-resistant *Salmonella*. In contrast, the lowest levels of MDR-NTS were recorded in the United States and Europe (9.6–22.6%) [7,8,9,10].

Vietnam, one of the leading countries in Southeast Asia in meat product manufacturing, ranks first in the prevalence of NTS, which is 2–5% in humans and 30–41% in retail meats and the farm environment [1]. Moreover, high rates of MDR NTS have emerged both in pigs (86%) and chickens (95%), pork and poultry meat production (40–75%), and in human sources (12.5–50%) [1,11,12,13]. Interestingly, the highest NTS prevalence in Vietnam was reported for duck and pig (94.3% and 91.3%) compared to chicken (64.7%) and exhibited a high resistance rate for tetracycline (58.5%), trimethoprim/sulfamethoxazole (58.1%), streptomycin (47.3%), ampicillin (39.8%), and chloramphenicol (37.3%) [12,13]. According to a study conducted by Coyne et al. (2019), four antibiotics (chlortetracycline, neomycin, colistin, and amoxicillin) considered crucial by the World Health Organization (WHO) for human infection treatment were found in pig and chicken feeds [14]. All this underlines the importance of localized antimicrobial resistance surveillance as well as careful monitoring of changes and distribution of drug-resistant microbial populations in animals and humans across this territory.

Based on whole-genome sequencing data, this study aims to provide genetic characteristics of multidrug-resistant non-typhoidal *Salmonella* from different sources with a focus on both antimicrobial resistance patterns and phylogenetic analysis. This research addresses the lack of data on multidrug-resistant salmonellosis in Southern Vietnam, a region underrepresented in the scientific literature compared to more extensively studied areas. Through a One Health approach, we provide a comprehensive examination of phenotypic and genotypic multidrug resistance patterns, with an emphasis on WGS-based serotype prediction and phylogenetic analysis of MDR *Salmonella* from livestock animals (pigs and chickens), retail meat (pork and broiler meat), and humans with acute diarrhea. This study elucidates the background of multidrug resistance, transmission pathways, population structure, and within-host diversity, potentially revealing local factors contributing to the selection, evolution, and spreading of multi-resistant *Salmonella* strains.

## 2. Results

### 2.1. NTS Serotype Identification

According to WGS analysis of 49 *Salmonella* enterica isolates, 14 different serotypes were identified: *S*. Typhimurium, *S*. Typhimurium monophasic, *S*. Indiana, *S*. London, *S*. Infantis, *S*. Rissen, *S*. Newport, *S*. Derby, *S*. Give, *S*. Enteritidis, *S*. Meleagridis, *S*. Kentucky, *S*. Panama, and *S*. Agona (Table 1). Five *S*. Typhimurium isolates were divided into three sequence types (STs), among which only ST34 belonged to a human source. All *S*. Typhimurium monophasic isolates belonged to the type ST34 without differences between host origin. Both *S*. Typhimurium and its monophasic variant were the dominant serotypes among human and pig sources and were identified in 72.7 and 44.4% of cases, respectively. All chicken samples belonged to *S*. Indiana ST17, whereas *S*. Infantis predominated in broiler meat samples (63.6%) and included two ST: one belonged to ST10, while ST32 represented the highest number of broiler meat isolates. All *S*. London ST155 isolates were detected in either pig or pork samples. The remaining serotypes (*S*. Newport, *S*. Derby, *S*. Give, *S*. Enteritidis, *S*. Meleagridis, *S*. Kentucky, *S*. Panama, and *S*. Agona) included one to two isolates predominantly from pig samples.

### 2.2. Antimicrobial Susceptibility Testing (AST) of NTS

The phenotypic AST results showed that among 49 *Salmonella* isolates, 48 (97.9%) were resistant to more than three antibiotic classes and were assigned as multidrug resistant (MDR), while four isolates (8.2%) were resistant to eight different antibiotic classes and were therefore defined as extensively drug resistant (XDR). All isolates (100%) were susceptible to only one of 15 antibiotics—MEM, whereas the highest percentage of resistance was observed against AMP and TET (45/49, 91.8%), followed by CIP/NAL and CHL (40/49, 81.6%, and 38/49, 77.6%, respectively).

The resistance rate for human isolates was highest for TET (11/11, 100%), followed by AMP, NAL/CIP, and CHL (10/11, 90.9% each) (Table 2). Similarly, *Salmonella* isolated from livestock and pork/broiler raw meat were most resistant to AMP, TET, CTX, NAL/CIP, and CHL. However, it is worth noting that isolates obtained from pigs have demonstrated a significantly higher resistance rate to CT (12/18, 66.7%) than those from other sources (χ^2^ = 17.479; df = 4; *p* = 0.002): pairwise comparison of the proportion of CT-resistant strains revealed differences between pig and chicken isolates (*p* = 0.01373) as well as pig and broiler meat isolates (*p* = 0.00039). A similar pattern was observed for broiler meat isolates, which showed the highest resistance to CTX (81.8%) compared to isolates from other sources (χ^2^ = 27.246; df = 4; *p* < 0.001): pairwise comparison of the proportion of CTX-resistant strains revealed differences between broiler meat and chicken isolates (*p* = 0.00481), broiler meat and humans (*p* = 0.00463), and broiler meat and pig isolates (*p* = 0.00001). All chicken isolates (100%) showed resistance to AMP and GEN/TOB, while 4/5 (80.0%) isolates were resistant to TET. Interestingly, none of the chicken and broiler isolates had similar resistance profiles.

We also analyzed antimicrobial resistance profiles of particular *Salmonella* serotypes combined with more than three isolates. For *S*. Typhimurium isolates and its monophasic variant, AMP and TET resistance rates were highest (94.1%), followed by NAL/CIP and CHL (88.2%). All *S*. Infantis isolates were resistant to β-lactams (except meropenem), NAL/CIP, TET, and GEN/TOB, whereas all *S*. Indiana and *S*. London isolates showed resistance to AMP, NAL/CIP, CHL, GEN/TOB, and AMP, NAL/CIP, CHL, and TMP/SMX, respectively. Among all *Salmonella* serotypes, only one *S*. Indiana isolate was resistant to AMI, while *S*. Infantis showed resistance to the third-generation cephalosporin CTX compared to other serotypes.

### 2.3. Correlation Between Phenotypic and Genotypic Antimicrobial Resistance of Salmonella Isolates

To investigate the relationship between phenotypic and genotypic antimicrobial resistance, we analyzed acquired resistance genes and/or resistance mutations for eight antibiotic classes using the ResFinder tool and assessed their correlation with the MIC pro-files of 49 *Salmonella* isolates (Table 3). Among all NTS isolates, 42 antimicrobial resistance genes (ARGs) have been identified. Overall, *Salmonella* isolates cultured from pig, chicken, and meat samples harbored more resistance genes compared to those from humans with acute diarrhea.

#### 2.3.1. Beta-Lactam Antibiotics

Phenotypic AST results demonstrated that among five β-lactam antibiotics, the majority (45/49, 91.8%) of NTS isolates were resistant to aminopenicillins (AMP: MIC ≥ 512 µg/mL), while 75% (37/49) showed susceptibility to third- and fourth-generation cephalosporins. All isolates were susceptible to carbapenems (MEM, MIC 0.008–0.047 mg/L) (not represented in Table 3). Twelve *Salmonella* isolates resistant to third- and fourth-generation cephalosporins (CTX with/or not CTZ and FEP) were isolated from all sources (human, pig, chicken, and broiler samples) and belonged to 4 serotypes—*S*. Infantis, *S*. Newport, *S*. Typhimurium monophasic, and *S*. London (Table 3).

All resistant phenotypes were confirmed by the presence of at least one gene from the beta-lactamases (BLs) group in the genome of *Salmonella* isolates. Among all gene types, *bla*TEM-1B was predominant (35/49, 71.4%), followed by *bla*CTX-Ms (12/45, 26.7%), *bla*OXA-1 (7/49, 14.3%), *bla*LAP-2 (3/49, 6.1%), and *bla*SHV-25 (1/49, 2.0%). Interestingly, the majority of resistant isolates (34/45, 75.6%) carried a single type of BL, among which the *bla*TEM-1B gene was predominantly found in isolates resistant only to AMP (24/33, 72.7%), whereas *bla*CTX-Ms prevailed in isolates resistant to cephalosporins. Two isolates from broiler and pork meat samples with the highest MIC values were positive for three *bla*CTX-M-55 + *bla*TEM-1B + *bla*LAP-2 BLs genes. Four isolates susceptible to β-lactams did not possess any BLs resistance genes.

#### 2.3.2. Aminoglycosides

Of 49 NTS isolates, 48 (97.9%) showed phenotypic susceptibility to AMI, while only 16 (32.7%) were susceptible to GEN and TOB (Table 3). All isolates from chicken samples and the vast majority of broiler isolates (10/11, 90.9%) were resistant to GEN/TOB. Isolates from pig and pork samples have demonstrated the lowest resistance to aminoglycosides.

Genotypically, this class of antimicrobials demonstrated the largest resistance diversity. In total, we identified 11 aminoglycoside-modifying enzyme (AME) genes: *aac(6′)-Iaa*, *aac(3)-IV*, *aac3IId*, *aac(6′)-lb-cr*, *aadA*, *aadB*, *aph(3′)-Ia*, *aph(6)-Id*, *aph(3″)-Ib*, *aph(4)-Ia*, and *rmtB*. One of the acetyl-transferase genes is *aac(6′)-Iaa*. Moreover, different combinations of genes, such as *aac(6′)-Iaa*, *aph(6)-Id*, *aph(3″)-Ib*, *aadA*, and *aadB*, were found in susceptible NTS isolates and were not associated with phenotypic resistance to GEN, TOB, and AMI in our study. The genetic profile in *Salmonella* isolates phenotypically resistant to GEN and TOB was marked by the presence *aac3IId* gene (which was predominantly detected in human and pig isolates) and *aac(3)-IV* and *aph(4)-Ia* (which prevailed in chicken and broiler meat isolates).

One isolate (*S*. Indiana) phenotypically resistant to all aminoglycosides with MIC > 256 µg/mL possessed seven resistance genes (*aac(6′)-Iaa* + *aac(3)-IV* + *aph(4)-Ia* + *aph(3″)*-*Ib* + *aph(6)-Id* + *aac(6′)-lb-cr* + *rmtB*) in its genome. Furthermore, the gene *rmtB*, which encodes for 16S rRNA methyltransferase, was detected specifically in this isolate and was absent in the other 48 *Salmonella* isolates.

#### 2.3.3. Quinolones

According to AST results, only 3 (6.1%) *Salmonella* isolates from human and pig samples were fully susceptible to quinolones—CIP and NAL (Table 3). Only one susceptible isolate did not have resistance genes. However, discrepancy between phenotypic and genotypic results was found for two isolates, assigned as quinolone-susceptible but carrying *aac(6′)-Ib-cr*, *parC*(T57S), and *qnrA1* resistance patterns. The *qnrS1* gene was the most frequent, present in 65.3% of resistant isolates. NAL- and CIP-resistant isolates with MIC > 256 µg/mL and MIC > 32 µg/mL, respectively, were characterized by the presence of double mutations D87Y/S83Y in the *gyrA* gene and T57S/S80R in the *parC* gene. It might also be noted that multiple resistance gene carriage in *Salmonella* was associated with resistance level to quinolones: the higher the MIC value, the more resistance genes were detected in *Salmonella* isolates. Both NAL- and CIP-resistant isolates with the highest MIC values had the most diverse genetic profiles and were associated with the following resistance patterns: *gyrA*(D87Y)/*gyrA*(S83Y), *parC*(T57S)/*parC*(S80R/I), *aac(6′)-Ib-cr*, and *qnrS1*.

#### 2.3.4. Other Antibiotics

We found that 16 out of 17 TMP/SMX-susceptible isolates carried *sul1*, *sul2*, and *sul3* resistance genes, whereas *dfrA12*, *dfrA14*, and *dfrA5* genes, involved in trimethoprim resistance development, were observed in only TMP/SMX-resistant isolate genomes.

Due to the lack of clinical breakpoints for NAL, CHL, AZM, and TET antimicrobials, ECOFF values were used to distinguish WT isolates (without phenotypically detectable resistance mechanisms) from NWT isolates with acquired resistance.

Of 49 *Salmonella* isolates, five (10.2%) were WT to CHL and did not harbor resistance genes in their genome. Genes *cmlA1*, *floR*, and *catA2*/*catA3*/*catB3* were associated with the NWT phenotype. All NWT isolates with MIC > 256 carried either the *floR* gene (encodes chloramphenicol acetyltransferase) or a combination of *floR* with *catA*/*B* genes. All chicken isolates were NWT to CHL and carried combinations of three resistance genes: *floR* + *cmlA1* + *catB3* and *floR* + *catA1*+ *catB3* (Table 3).

Overall, the majority of NTS isolates (38/49, 77.6%) exhibited a WT phenotype to AZM, and no resistance genes were found in their genome. In contrast, NWT isolates predominantly carried the phosphotransferase *mph(A)* gene (10/11, 90.9%); one isolate contained the macrolide efflux protein *mef(B)* gene.

Among 49 NTS, 33 (67.3%) isolates were phenotypically susceptible to colistin, and no resistance genes were found in their genome. Resistance to CT was associated with three variants of the mobile colistin resistance *mcr* gene (1.1, 3.1, 3.5). Interestingly, all isolates from chicken and broiler meat samples were susceptible to CT, whereas the majority of pig isolates (12/18, 66.7%) showed resistance to CT.

Four isolates (8.2%) had a WT phenotype to TET; two of them contained the *tetM* gene. NWT isolates harbored either *tetA*, *tetB*, or combinations of *tetA* + *tetB* and *tetA* + *tetM* genes.

### 2.4. Plasmid Analysis

**Figure 1:**
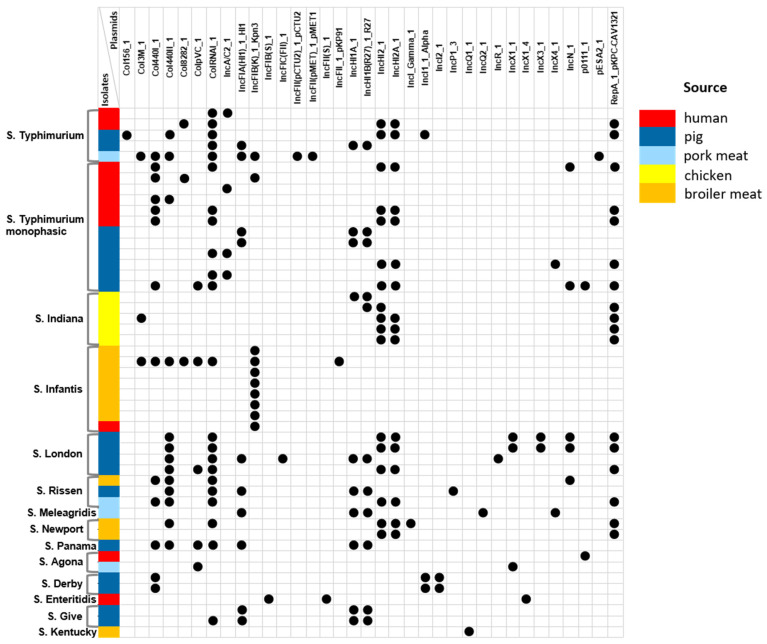
Plasmid profiling in the Vietnamese NTS isolates from different sources.

Using PlasmidFinder 2.1 software, we identified 35 AMR plasmids among 49 NTS isolates: 25 different incompatibility (Inc) family plasmids were detected in 48/49 (97.9%) isolates, while the colicin (Col) group plasmids were contained within 28/49 (57.1%) isolates (Figure 1).

The most frequently detected incompatibility groups were IncHI2_1 and IncHI2A_1, found in 17 (34.7%) NTS isolates from various serovars and sources. Both plasmids were associated with the plasmid RepA_1_pKPC-CAV1321. Other replicon types, IncHI1A_1 and IncHI1B(R27)_1_R27, were detected in 10 (20.4%) isolates, mostly from pigs and pork meat (9/10, 90.0%). Interestingly, the IncFIA(HI1)_1_HI1 replicon was carried by 11 *Salmonella* strains, of which 10 (90.9%) were also isolated from either pig or pork meat samples. The plasmid replicon IncFIB(K)_1_Kpn3, on the contrary, was predominant among isolates from broiler meat (70.0%) and was harbored by all *S*. Infantis isolates. Notably, many previous studies reported that the IncFII(S) plasmid was one of the most widely represented types and was present in all clinical *Salmonella* isolates [15,16]. Our results are inconsistent with these findings and showed that only one human *S*. Enteritidis isolate carries the IncFII(S)_1 replicon. Interestingly, only two *S*. London isolates from pigs presented the IncX3_1 plasmid (primarily responsible for the dissemination of antibiotic resistance genes) alongside the IncX1_1 and IncN_1 plasmid types.

Among the colicin family plasmids, ColRNAI_1 was the most frequently detected across all *S*. London, *S*. Rissen, and *S*. Typhimurium isolates, irrespective of source. Another Col440I_1 plasmid was detected in 28.6% of NTS isolates regardless of serotype and source, whereas Col440II_1 and ColpVC_1 plasmids were the most frequently observed in pig and pork meat isolates (9/13, 69.2%, and 4/5, 80.0%, respectively). All *S*. London and *S*. Rissen isolates contained ColRNAI_1 along with the Col440II_1 plasmid, while one *S*. Infantis broiler meat isolate presented five types of Col plasmids (Col3M_1, Col440I_1, Col440II_1, Col8282_1, ColpVC_1, and ColRNAI_1). Notably, six types of Col plasmids were detected in broiler isolates, whereas only one Col440I_1 plasmid was detected among chicken isolates.

### 2.5. Phylogenetic Analysis

**Figure 2:**
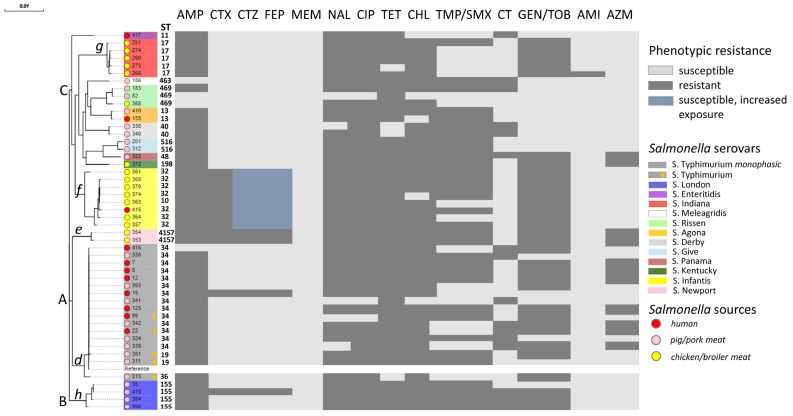
Maximum likelihood phylogenetic tree of 49 Vietnamese NTS isolates from different sources based on core SNVs with respect to phenotypic resistance profile. A, B, C and *d*, *e*, *f*, *g*, *h* stand for clusters and subclusters, respectively. AMP—ampicillin, CTZ—ceftazidime, CTX—cefotaxime, FEP—cefepime, MEM—meropenem, NAL—nalidixic acid, CIP—ciprofloxacin, TET—tetracycline, CHL—chloramphenicol, TMP/SMX—trimethoprim/sulfamethoxazole, CT—colistin, GEN—gentamycin, TOB—tobramycin, AMI—amikacin, AZM—azithromycin.

Given that MLST (Multi Locus Sequence Typing) can not sufficiently differentiate *Salmonella* serotypes, for phylogenetic analysis we used core genome SNPs as having the highest resolution compared to MLST, core genome MLST, and whole-genome MLST [17]. Based on core SNPs of 49 *Salmonella* genomes, we constructed the maximum likelihood phylogenetic tree with respect to the phenotypic antibiotic resistance profile (Figure 2).

As a result, all 49 NTS isolates clustered according to the ST serotypes and formed three distinct phylogenetic clusters: A, B, and C. The most predominant cluster A was divided into two subclusters, *d* and *e*. Subcluster *d* comprised *S*. Typhimurium isolates and its monophasic variant; however, within this subcluster, ST34 and ST19 isolates (including reference ST19 strain) formed a separate group adjacent to the ST36 isolate. Subcluster *e* included *S*. Newport ST4157 isolates. *S*. London ST155 isolates grouped together and formed Cluster B. Cluster C combined 10 different *Salmonella* serotypes, among which a single *S*. Enteritidis ST11 from a human formed a distant subcluster. Subclusters *f* and *g* were formed by *S*. Infantis ST32/ST10 and *S*. Indiana ST17 strains, isolated predominantly from chicken and broiler meat samples. The remaining groups of subclusters contained one to three *Salmonella* isolates.

Notably, each *Salmonella* serotype in this analysis formed a distinct cluster or subcluster, whereas the phylogenetic tree showed close genetic relatedness between strains isolated from different sources. These observations indicate that the clustering of NTS isolates is significantly affected by serotypes rather than isolation source. Moreover, since *Salmonella* strains exhibit different mechanisms of genomic diversity and transmission pathways, obtained results suggest the possibility of NTS transmission across different hosts and locations, ultimately leading to the foodborne illness in humans.

**Figure 3:**
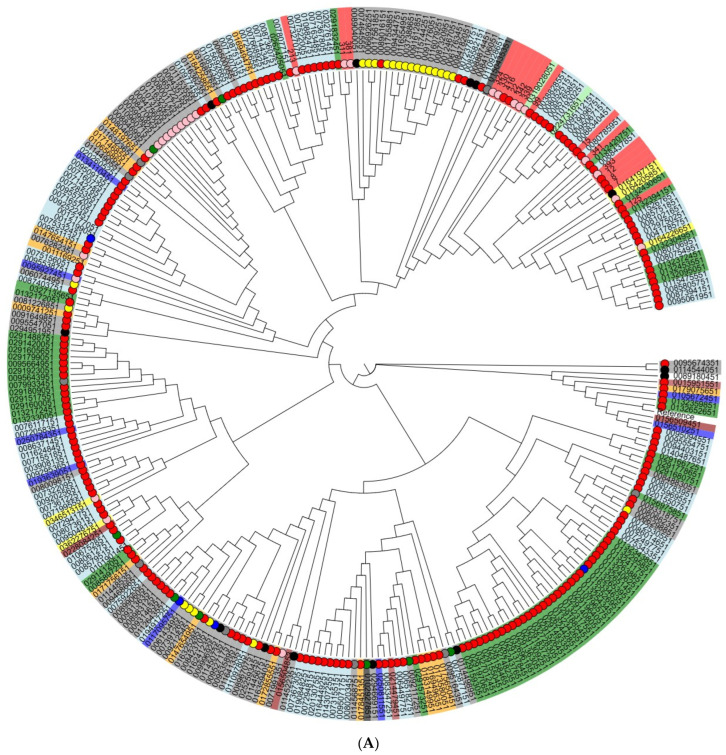

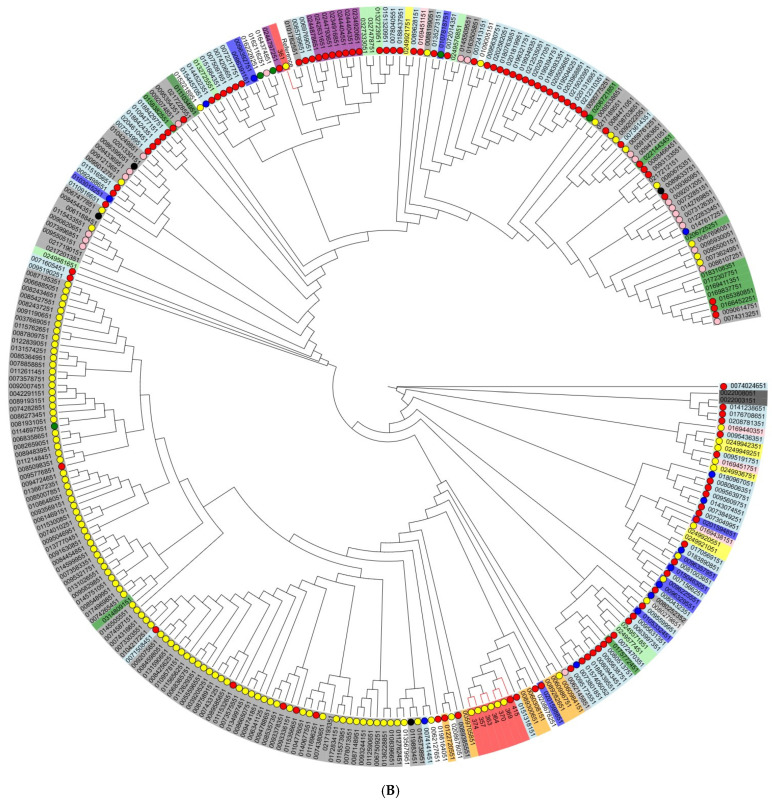
Phylogenetic (cladogram) tree based on core-genome single nucleotide polymorphisms (SNPs) of (**A**) *S*. Typhimurium (and its monophasic variant) isolates and (**B**) *S*. Infantis isolates. The cladogram was created using the maximum-likelihood algorithm. Small colored circles innermost stand for the sources of NTS isolates: red—human, pink—porcine, yellow—poultry, black—bovine, and green—food; the absence of a circle indicates isolates with unknown origin. The countries of origin of *Salmonella* isolates are marked by color in rectangles: (**A**) green: Australia; orange: Canada; yellow: China; light green: Denmark; dark red: Japan; light blue: United Kingdom; grey: USA; red: Vietnam; blue: others. (**B**) purple: South Africa; light green: Australia; white: Brazil; green: Canada; blue: Germany; pink: Hungary; yellow: Slovenia; orange: Peru; light blue: United Kingdom; grey: USA; red: Vietnam; white: others.

To assess the genetic diversity and relationships between Vietnamese isolates from different sources, we generated cladograms based on core-genome SNPs for two serotypes that predominated in our investigation: *S*. Infantis and *S*. Typhimurium (and its monophasic variant) isolates (Figure 3). In total, for this analysis, we used 292 *S*. Infantis and 283 *S*. Typhimurium genomes of the corresponding serotypes from different sources, regions, and years, randomly selected from the international GenBank database.

As a result, seven of eight Vietnamese *S*. Infantis isolates from both broiler meat and humans were clustered together in one subcluster; interestingly, these isolates showed a close genetic relationship with human isolates from the United Kingdom. The majority of Vietnamese *S*. Typhimurium isolates did not completely cluster together; however, irrespective of pig or human source, they were included in one subcluster with those isolated predominantly from humans. Two *S.* Typhimurium isolates from pork meat had a close relationship with isolates from poultry samples obtained in the USA.

## 3. Discussion

Since WGS has been proven to be the most reliable, accurate, and effective method for serovar and sequence type identification, as well as antimicrobial resistance patterns and phylogenetic relationships estimation between different isolates, we used this approach for the comprehensive characterization of *Salmonella* isolates from different sources in southern regions of Vietnam.

According to our study, monophasic *S.* Typhimurium ST34 [4: i: -] (one of the most common in Vietnam) was the predominant serotype found among pigs and patients with diarrhea [13,18]. Moreover, a high prevalence of monophasic *S.* Typhimurium in pig farms was detected in northern and central Vietnam. Recent studies have shown that the highest similarity is observed between human and pig serovars [19]. Our phylogenetic analysis showed that monophasic *S.* Typhimurium isolates from humans and pigs clustered together into the same clades, which implies the transmission of this pathogen. *S.* London was typical for both pig and pork samples, while *S.* Derby and *S.* Give were only typical for pig samples. Taking into account that pork is the most consumed meat in Vietnam and has the highest (91.3%) NTS prevalence among farm livestock products, the pig production sector poses one of the main reservoirs for human infection [13,20]. All chicken samples in our study belonged to the *S.* Infantis serotype, while *S.* Indiana was mostly found in broiler meat samples. It has been shown that *S.* Indiana is a relevant serotype associated with chicken meat in Vietnam and exhibited resistance to four to six classes of antimicrobials [21]. However, according to the study by Ta et al. (2014), *S.* Indiana was the fifth most frequently identified serotype in retail raw poultry meat in Vietnam after Albany, Agona, Dabou, and Hadar [22]. The variety of *Salmonella* serotypes may reflect the geographical, collection time, and identification methods diversity.

In the current study, we deliberately included NTS isolates that were resistant to more than three antibiotic classes and assigned as MDR. The spread of MDR bacteria in humans, the livestock sector, veterinary medicine, and ready-to-eat products is an alarming public health concern and affects not only humans but also animals, causing emergency situations worldwide. Overall, data from all regions of Vietnam show an increase in AMR and MDR over time. A study from 2000–2020 reported an increasing trend in the pooled AMR prevalence in NTS for quinolones (15.6%), 3rd-, 4th-, and 5th-generation cephalosporins (23.7%), tetracyclines (12.9%), amphenicol (17.8%), and multidrug resistance (11.4%) [1].

Our results demonstrated similarity in phenotypic resistance profiles between different serotypes, except that *S.* Infantis showed the highest resistance to third- and fourth-generation cephalosporins compared to others. Despite the MDR status, all NTS isolates were susceptible to meropenem—a beta-lactam antibiotic of the carbapenem class—which has been proven efficacious as monotherapy in the treatment of a broad spectrum of infections as well as those caused by organisms resistant to other antimicrobials. On the contrary, the highest resistance was observed against fluoroquinolones—CIP and NAL—which are among the highest priority critically important antimicrobials (HPCIAs) and the drugs of choice for salmonellosis treatment. Notably, among all NTS isolates, the highest MIC values showed chicken isolates *S.* Indiana ST17, which was recognized as an epidemiologically important type due to its high resistance level to most widely used classes of antimicrobials [23]. Moreover, the majority of pig isolates have demonstrated significantly higher MICs to another class of HPCIAs—polymyxin, represented by CT in our study. This finding is in line with other studies from Northern Vietnam: an NTS survey (*n* = 138) in pigs (2011–2012) revealed phenotypic resistance to CT in only four *S*. Typhimurium isolates (2.9%), while subsequent surveillance data (2013–2019) showed a CT resistance rate of 9–26%, predominantly in isolates from pigs, retail chickens, and pork [1,13]. The resistance rate for *Salmonella* isolates from humans as well as those from animals and pork/broiler raw meat was highest for tetracycline, beta-lactams, phenicol, and quinolones. This important observation is consistent with the previous study by Nhung et al. (2017), which found that sulfonamides, tetracyclines, and macrolides were the most frequently detected residues in pig and poultry production in Vietnam [24]. This finding indicates not only poor sanitation in the small-scale livestock farms but also limited surveillance under inadequate usage of antibiotic agents in veterinary or medicated feed, boosting the risk of selecting multi-resistant strains and transferring them from animals to humans, which, after all, leads to a negative impact on the population’s health in general. However, so far Vietnam does not have a nationwide surveillance system to monitor NTS and resistance level to AMR in human and livestock populations [1].

It is well known that the presence of AMR genes is responsible for *Salmonella* resistance development. In our study, all NTS-resistant and NWT had at least one resistance gene in their genome. Moreover, the number of resistance genes was associated with resistance level: the higher the MIC value, the broader the genetic profile detected in *Salmonella* isolates. This observation suggests that high antibiotic concentrations promote selective pressure on cooperative genes, implying that multiple genes or sequential mutations in AMR genes are required for antibiotic resistance to develop. The highest correlation between phenotypic and genotypic resistance was found for azithromycin, chloramphenicol, and ampicillin (100%) and above 90 and 95% for tetracycline and colistin, respectively. However, it is worth noting that for four antimicrobial classes (quinolones, aminoglycosides, sulfonamides, and tetracycline), AMR gene content did not match the phenotype: the presence of one to four resistance genes was common for both susceptible/WT and resistant/NWT isolates. This discrepancy may indicate either a lack of expression in the absence of antibiotic exposure conditions or a low level of resistance genes for high-level clinical resistance development, since *Salmonella* resistance is polygenic [23].

The *bla*TEM-1B resistance gene has predominated among β-lactam-resistant isolates, while *bla*CTX-M-55 and *bla*CTX-M-65 genes (or co-harboring *bla*CTX-M-55/*bla*CTX-M-65 with *bla*TEM-1B) were associated with resistance to cephalosporins. There are many β-lactamase variants disseminated in *Salmonella* isolates globally; however, among Vietnamese isolates, we revealed quite a narrow spectrum of BL genes, and the majority of resistant isolates (75.6%) carried only one gene, which is in line with a prior study from Vietnam [25]. Moreover, *bla*CTX-M-producing isolates were mostly identified from broiler meat samples and showed the highest resistance to cephalosporins. Previous studies have demonstrated the high prevalence of *Salmonella* in broiler meat in Vietnam, ranging from 69 to 77%, depending on the region [26]. Since *bla*CTX-Ms are often cross-resistant to other various antibiotics and mobilize to all major plasmids approximately 10 times more frequently than other class A β-lactamases, *bla*CTX-Ms-harboring *Salmonella* in broiler meat represent a troubling and intractable issue for human and food safety [27].

Fluoroquinolone resistance in NTS isolates from Vietnam was attributed not only to point mutations in the quinolone resistance-determining regions (QRDR), involving chromosomal *gyr* and *par* topoisomerase genes, but also to plasmid-mediated quinolone resistance (PMQR) genes *qnr* and *aac(6′)-lb-cr*, encoding pentapeptide topoisomerase-binding proteins and quinolone-modifying enzymes, respectively. The low-level resistance to CIP was mediated by the presence of *qnr*S1 and *aac(6′)-lb-cr* PMQR genes, whilst double mutations S83F/D87N in *gyrA* and T57S/S80I in *parC* QRDR genes were related to high resistance levels to both NAL and CIP. Our results are in agreement with other studies suggesting that high-level quinolone resistance results from the gradual accumulation of QRDR mutations [28,29,30,31]. Compared with the highest cephalosporin resistance in broiler meat isolates, the highest resistance to quinolones was demonstrated by *S.* Indiana isolates from chicken, which had the most diverse genetic resistance profile and harbored both QRDR and PMQR genes.

*Salmonella* isolates resistant to aminoglycosides exhibited the most complex and diverse genetic profile compared to those resistant to other classes of antimicrobials. Four families of aminoglycoside-modifying enzyme (AME) genes were identified in this study: aac, *aph*, *aad*, and *rmt*, among which aminoglycoside acetyltransferase *aac(6′)-Iaa* was predominant and possessed by all 49 NTS isolates regardless of phenotypic resistance-susceptibility status. Notably, nucleotidyltransferase ant genes associated with enzymatic inactivation of aminoglycosides were not detected in our sample. Among the entire spectrum of AME genes, only *aac3IId*, *aac(3)-IV*, *aph(4)-Ia*, and *aac(6′)-lb-cr* genes were associated with resistance to GEN and TOB. Since the *aac(6′)-Ib-cr* gene is known to be involved in cross-resistance to aminoglycosides and fluoroquinolones, five chicken isolates harboring this gene were resistant to CIP/NAL and GEN/TOB antibiotics. Moreover, among 49 NTS isolates, only one chicken isolate was resistant to all aminoglycosides (GEN, TOB, and AMI) and was characterized by the presence of the 16S rRNA methylase *rmtB* gene in its genetic profile, which has been shown to have a low prevalence in *Salmonella* and confers resistance to multiple aminoglycosides [32,33].

The recent emergence of colistin resistance and the rapid transmission of the mobile colistin resistance gene (*mcr*) among different bacterial species worldwide raise concerns about the uncontrolled transmission of colistin-resistant bacteria across all ecological niches [34]. Our study demonstrated that all isolates from chicken and broiler meat were susceptible to colistin, whilst NTS from pig isolates showed the highest resistance (66.7%) compared to human (27.3%) and pork meat (25%) isolates. Among all *mcr* gene variants (*mcr-1* to *mcr-10*), only *mcr-1* and *mcr-3* variants were detected in CT-resistant isolates. Similar results were obtained in another study from Vietnam that showed the widespread use of colistin or colistin-containing feeds in livestock breeding: more than 70% of pig and chicken fecal samples contained CT-resistant *E. coli* with *mcr-1* or *mcr-3* genes [1]. Interestingly, one human and three pig isolates showed resistance to CT; however, without association with the plasmid-borne *mcr* genes. This suggests the presence of other underlying resistance mechanisms for *Salmonella* to develop colistin resistance, such as efflux pumps, chromosomal mutations, and/or alterations in the O-antigen polymerase gene [35]. Thus, increased attention must be directed towards monitoring CT-resistant bacteria and excessive antimicrobial utilization in the agricultural sector.

In this study, high rates of NWT-phenotype to chloramphenicol and tetracycline (~90 and 92%) were observed for NTS serovars from different sources. The genetic profile for CHL-NWT isolates was related to chloramphenicol acetyltransferases *cat* genes types A/B, as well as chloramphenicol exporters *cmlA* and *floR* efflux pump genes. Despite the general observation that the presence of more than one phenicol resistance gene in the same *Salmonella* isolate is rare, in our study, 48% of the Vietnamese CHL-resistant isolates contained two and three phenicol resistance genes each [36]. So far, among the wide variety of tetracycline resistance genes, only five—*tetA*, *tetB*, *tetC*, *tetD,* and *tetG*—have been reported for *Salmonella* [32]. Intriguingly, we found another ribosomal protection gene, *tetM*, co-carried with the *tetA* gene in 20% of TET-NWT isolates, predominantly from pigs. It is well known that the *tetM* gene is usually carried by *Enterococcus* sp., is atypical for Gram-negative bacteria, and was first described in *E. coli* strains from chickens and pigs [36,37]. Subsequently, Jurado-Rabadán S. et al. (2014) identified the *tetM* gene in doxycycline-resistant *E. coli* isolates from pigs [38]. Taken together with our results, these data suggest that the presence of the *tetM* gene in porcine *Salmonella* isolates may be the result of possible horizontal transfer of this gene from intestinal enterococci or *E. coli* [37,38].

Vietnamese NTS isolates exhibited a relatively high prevalence of resistance (65%) to trimethoprim/sulfamethoxazole, with the highest rates observed in isolates from pigs and pork meat. However, we found that sulfonamide resistance *sul1*/*sul2*/*sul3* genes by themselves were present only in TMP/SMX-susceptible isolates, while the combination of *sul* genes with transferable dihydrofolate reductase *dfrA* was typical exclusively for TMP/SMX-resistant isolates. Thus, according to our findings, the presence of *dfrA* genes is responsible for the development of TMP/SMX resistance. On the other hand, this is contrary to previous studies, which have suggested that TMP/SMX resistance in *Salmonella* is typically linked to the presence of either *sul1* or *sul2* genes [39]. Notably, for phenotypic testing we used a synergistic TMP and SMX combination, which does not allow us to determine whether the *dfrA* gene alone is sufficient to confer resistance to TMP/SMX.

One of the most effective antimicrobials in our study was azithromycin, to which *Salmonella* isolates showed a low NWT-phenotype rate (25%) with a strong association of phenotypic resistance with the macrolide 2′-phosphotransferase *mph(A)* and efflux pump *mef(B)* genes. Given that macrolides remain one of the mainstays of treatment for MDR in countries with a high burden of fluoroquinolone resistance, the spread of azithromycin resistance among circulating strains could have a major impact on the dwindling options for treatment strategies.

The present study has several limitations. First, the sample size is rather small and may not reflect the overall resistance patterns in Vietnam. Second, serotyping analysis classified the 49 NTS isolates into 14 different serotypes, resulting in an imbalance of *Salmonella* serotypes and source categories that tend to be statistically fragile. Third, due to the limitations of short-read sequencing technology, we were unable to precisely determine the location of resistance genes on the chromosome or plasmids. However, despite these limitations, our data provided relevant information on the genetic patterns of multidrug-resistant *Salmonella* isolates from different sources and highlighted the need for a controlled approach to antibiotic overuse in animal husbandry.

## 4. Materials and Methods

### 4.1. Study Design, Sampling Collection, and Bacterial Isolation

This study was performed within a framework of scientific cooperation between the St. Petersburg Pasteur Institute, Russia, and the Pasteur Institute in Ho Chi Minh City, Vietnam. A total of 49 isolates from pigs (*n* = 18) and chickens (*n* = 5) from family-run livestock farms, pork (*n* = 4) and broiler (*n* = 11) meat from retail outlets, and humans (*n* = 11) with acute diarrhea were collected during 2012–2021 from Ho Chi Minh City (*n* = 20), Dong Nai (*n* = 24), Long An (*n* = 1), Ben Tre (*n* = 1), and An Giang (*n* = 3) provinces, Vietnam.

*Salmonella* strains were isolated according to the International Standard ISO 6579-1:2017, using buffered peptone water (pre-enrichment), tetrathionate broth, and Rappaport Vassiliadis soy peptone broth (selective enrichment), followed by subcultivation onto XLD agar plates and Hektoen Enteric agar plates (Condalab, Madrid, Spain) [40]. The isolated bacteria were identified to a species level with MALDI-TOF MS (Autof MS1000, Zhengzhou, China). The isolated strains were stored in cryotubes with beads for microorganisms (DELTALAB, S.L., Barcelona, Spain) at −70 °C. *Salmonella* serotypes and appropriate sequence types (STs) were determined by whole-genome sequencing data.

### 4.2. Phenotypic Antimicrobial Susceptibility Testing (AST)

Phenotypic antimicrobial susceptibility testing (AST) was performed according to European Committee on Antimicrobial Susceptibility Testing (EUCAST) recommendations by the serial broth microdilution testing using “MPC-MICRO PS” strips (St. Petersburg Pasteur Institute, Russia) with Muller Hinton broth (Condalab, Madrid, Spain) and gradient diffusion method using Muller Hinton agar (Condalab, Madrid, Spain) with the following antibiotics: β-lactams (ampicillin (AMP)); cephalosporins (ceftazidime (CTZ), cefotaxime (CTX), cefepime (FEP)); carbapenem (meropenem (MEM)); quinolones (nalidixic acid (NAL), ciprofloxacin (CIP)); tetracycline (TET); phenicol (chloramphenicol (CHL)); trimethoprim/sulfamethoxazole (TMP/SMX); polymyxins (colistin (CT)); aminoglycosides (gentamycin (GEN), tobramycin (TOB), amikacin (AMI)); and macrolides (azithromycin (AZM)). The results were interpreted according to the European Committee on Antimicrobial Susceptibility Testing (EUCAST) clinical breakpoint interpretation (EUCAST, 2024). In some cases where clinical breakpoints were not available, epidemiological cut-off values (ECOFF) were used to distinguish wild-type (WT) isolates from isolates with acquired resistance (non-wild-type, NWT): NAL (8 mg/mL), TET (8 mg/mL), CHL (16 mg/mL), AZM (16 mg/mL). *Salmonella* isolates showed resistance to three or more different classes of antimicrobials and were defined as multidrug resistant (MDR).

### 4.3. DNA Extraction and Sequencing

Genomic DNA of all 49 *Salmonella* isolates was isolated by the QIAamp DNA Mini Kit (QIAGEN GmbH, Hilden, Germany) according to the manufacturer’s guidelines. The DNA concentration was quantified on a Qubit 4.0 fluorometer. Whole-genome shotgun DNA libraries were prepared using the MGIEasy FS DNA Library Prep Set and then sequenced on a DNBSEQ-G50 sequencer (MGI Tech Co. Ltd., Beijing, China) in a 2 × 100 paired-end (PE) mode.

### 4.4. Bioinformatics Analysis

The raw paired-end reads were analyzed using FastQC software (v.0.12.1; Babraham Institute, Cambridge, UK), then trimmed and filtered by Trim Galore! (version 0.6.7). Bacterial genomes were assembled de novo by SPAdes assembler software (version 3.13.1), and the results were evaluated by QUAST (version 5.2.0). The assembled genome sequences for all isolates were deposited to the NCBI GenBank under the project number PRJNA933104.

Core-genome SNPs were called using Snippy pipeline v.4.6.0 (https://github.com/tseemann/snippy, accessed on 16 June 2022). *Salmonella enterica* subsp. *enterica* serovar Typhimurium str. LT2 was used as a reference genome (GenBank accession number AE006468.2). Mutation clustering analysis, maximum likelihood tree, and cladograms were constructed using the RAxML-NG inference tool based on the core genome SNPs obtained using the Snippy core. To analyze the phylogeographic distribution of *Salmonella* isolates from Vietnam, 34,291 assembled *Salmonella* genomes were downloaded from the NCBI Genome database (Nucleic Acids Research; accessed 1 January 2013; http://www.ncbi.nlm.nih.gov/sites/genome) as available in February 2024. By metadata filtering, 575 *Salmonella* Infantis and Typhimurium genomes were randomly selected for further investigation and added to the phylogenetic analysis. The reliability of the phylogenetic tree branches was evaluated by the bootstrapping method (1000 replications). Phylogenetic trees and cladograms were visualized by Evolview v3 (https://www.evolgenius.info/evolview/, accessed on 22 May 2019).

Additionally, the ResFinder 4.0 database with default parameters was used for antimicrobial resistance gene (ARG) detection. The SeqSero 1.2 service was used for *Salmonella* serotype prediction based on WGS information (Center for Genomic Epidemiology, https://www.genomicepidemiology.org/, accessed on 11 August 2020). For plasmid detection, PlasmidFinder 2.1 was used (https://github.com/genomicepidemiology/plasmidfinder, accessed on 31 January 2021).

### 4.5. Statistical Analysis

All statistics were performed in the R programming language, environment version 4.3.1. A *p*-value < 0.05 was defined as statistical significance. The data were tested by the chi-square (χ^2^) test and Fisher’s exact test.

## 5. Conclusions

Using a WGS-based approach, we conducted a comprehensive investigation of non-typhoidal *Salmonella* serovars from different sources in Southern Vietnam with a particular focus on multidrug resistance patterns. This study showed that both livestock animals and meat products are a major source of *Salmonella* with high levels of resistance to multiple antimicrobials and an expanded spectrum of resistance genes and plasmids. Despite the limited number of samples, we were able to determine that NTS strains isolated from poultry and pigs exhibit resistance to the highest priority antimicrobials—quinolones and β-lactams—which are essential for severe salmonellosis treatment. Additionally, these strains showed resistance to aminoglycosides, tetracyclines, and sulfonamides. The obtained results highlight the utmost importance of issues related to the selection, spreading, and transmission of multi-resistant strains along the entire meat production chain from animals to humans. As the level of multidrug-resistant NTS in Vietnam is steadily increasing, this should serve as an incentive to develop strict preventive control strategies and food safety regulations, along with more stringent and enhanced surveillance under sanitation practices and antibiotic overuse in the animal husbandry sector. Thus, ongoing monitoring of antimicrobial resistance patterns in different *Salmonella* serovars among humans, animals, and meat products is a prerequisite for efficient control of genome dynamics, NTS diversity, prevalence, and relationships between different hosts.

## Figures and Tables

**Table 1:** Serotyping results of 49 NTS isolated from different sources.

Serotype	ST	Number	Source	Place and Year of Isolation
*S.* Typhimurium monophasic (*n* = 12)	34	6	human	Ho Chi Minh city, 2012 (*n* = 1); Dong Nai, 2013 (*n* = 4), Ben Tre, 2018 (*n* = 1)
		6	pig	Dong Nai, 2012 (*n* = 2), 2021 (*n* = 4)
*S.* Typhimurium (*n* = 5)	34	2	human	Dong Nai, 2012, 2013
	36	1	pig	Ho Chi Minh city, 2014
	19	1	pork	Ho Chi Minh city, 2021
		1	pig	Dong Nai, 2012
*S.* Indiana (*n* = 5)	17	5	chicken	Dong Nai, 2012
*S.* London (*n* = 4)	155	2	pig	Dong Nai, 2022
		2	pork	Ho Chi Minh city, 2014, 2021
*S.* Infantis (*n* = 8)	32	1	human	An Giang, 2021
		6	broiler meat	Ho Chi Minh city, 2021
	10	1	broiler meat	Ho Chi Minh city, 2021
*S.* Rissen (*n* = 3)	469	1	pig	Ho Chi Minh city, 2014
		1	pork	Long An, 2013
		1	broiler meat	Ho Chi Minh city, 2021
*S.* Newport (*n* = 2)	4157	2	broiler meat	Ho Chi Minh city, 2021
*S.* Derby (*n* = 2)	40	2	pig	Dong Nai, 2021
*S.* Give (*n* = 2)	16	2	pig	Dong Nai, 2021 Ho Chi Minh city, 2014
*S.* Enteritidis (*n* = 1)	11	1	human	An Giang, 2020
*S.* Meleagridis (*n* = 1)	463	1	pork	Ho Chi Minh city, 2014
*S.* Kentucky (*n* = 1)	198	1	broiler meat	Ho Chi Minh city, 2021
*S.* Panama (*n* = 1)	48	1	pig	Dong Nai, 2012
*S.* Agona (*n* = 2)	13	1	human	An Giang, 2021
		1	pig	Ho Chi Minh city, 2014

**Table 2:** Antimicrobial resistance of 49 NTS isolates from different sources. Non-wild-type isolates were considered as resistant to NAL, TET, CHL, and AZM.

Antimicrobials	Resistant Isolates, *n* (%)					
	Human *n* = 11	Pig *n* = 18	Chicken *n* = 5	Broiler Meat *n* = 11	Pork Meat *n* = 4	Total *n* = 49
Ampicillin	10 (90.9)	17 (94.4)	5 (100)	10 (90.9)	3 (75.0)	45 (91.8)
Cefotaxime	2 (18.2)	0	0	9 (81.8)	1 (25.0)	12 (24.5)
Ceftazidime	1 (9.1)	0	0	2 (18.2)	1 (25.0)	4 (8.2)
Cefepime	1 (9.1)	0	0	2 (18.2)	1 (25.0)	4 (8.2)
Nalidixic acid	10 (90.9)	15 (83.3)	5 (100)	11 (100)	3 (75.0)	44 (89.8)
Ciprofloxacin	10 (90.9)	17 (94.4)	5 (100)	11 (100)	3 (75)	46 (93.9)
Tetracycline	11 (100)	15 (83.3)	4 (80.0)	11 (100)	4 (100)	45 (91.8)
Chloramphenicol	10 (90.9)	16 (88.9)	5 (100)	10 (90.9)	3 (75.0)	44 (89.8)
Trimethoprim/Sulfamethoxazole	7 (63.6)	13 (72.2)	1 (20.0)	8 (72.7)	3 (75.0)	32 (65.3)
Colistin	3 (27.3)	12 (66.7)	0	0	1 (25.0)	16 (32.7)
Gentamycin/Tobramycin	8 (72.7)	7 (38.9)	5 (100)	10 (90.9)	2 (50.0)	32 (65.3)
Amikacin	0	0	1 (20.0)	0	0	1 (2.0)
Azithromycin	5 (45.5)	3 (16.7)	0	3 (27.3)	0	11 (22.4)

**Table 3:** Correlation between genotypic and phenotypic AST and comparison of the resistance profile of NTS isolates from different sources.

Genotype		MIC. µg/mL				Source				
						Human (*n* = 11)	Pig (*n* = 18)	Chicken (*n* = 5)	Broiler Meat (*n* = 11)	Pork Meat (*n* = 4)
		AMP (R > 8)	CTX (R > 2)	CTZ (R > 4)	FEP (R > 4)					
No genes		1–8	0.047–0.125	0.047–0.125	0.016–0.19	1	1		1	1
OXA-1		>512	0.125–0.19	0.19–0.25	0.25–0.38	1	1			
TEM-1B		>512	0.047–0.25	0.125–1	0.032–0.25	6	16		1	1
TEM-1B + SHV-25		>512	0.94 *	0.38	0.064					1
TEM-1B + OXA-1		>512	0.125–0.25	0.25–0.5	0.38–1			5		
TEM-1B + LAP-2		>512	0.25	0.38	0.19	1				
CTX-M-65		>512	>32	1.5–2	2–4	1			6	
CTX-M-65 + TEM-1B		>512	>32	2	3				1	
CTX-M-55		>512	>32	256	48	1				
CTX-M-55 + TEM-1B		>512	>32	24	24				1	
CTX-M-55 + TEM-1B + LAP-2		>512	>32	16–256	16–128				1	1
		**GEN (R > 2)**	**TOB (R > 2)**	**AMI (R > 8)**						
*aac(6′)-Iaa + aph(6)-Id*		0.75	0.75	2					1	
*aac(6′)-Iaa + aph(3″)-Ib + aph(6)-Id*		0.25–0.75	0.5–1.5	1.5–2		3	2			0
*aac(6′)-Iaa + aadA*		0.5–1	0.5–1.5	1.5–3			7			1
*aac(6′)-Iaa + aph(3″)-Ib + aph(6)-Id + aadA*		0.5–1	1	2			2			
*aac(6′)-Iaa + aadA + aadB*		0.75	1.5	3						1
*aac(6′)-Iaa + aac3IId + aadA*		3–256	3–6	2			1			1
*aac(6′)-Iaa + aac3IId + aph(3′)-Ia + aadA*		4–48	3–4	2			2			
*aac(6′)-Iaa + aac3IId + aph(3″)-Ib + aph(6)-Id*		32–64	3–4	1.5–3		2	2			
*aac(6′)-Iaa + aac3IId + aph(3″)-Ib + aph(6)-Id + aadA*		257	16	2		1				
*aac(6′)-Iaa + aac3IId + aph(3′)-Ia + aph(6)-Id + aadA*		24–32	3–6	1.5–2					1	1
*aac(6′)-Iaa + aac3IId + aph(3′)-Ia + aph(3″)-Ib + aph(6)-Id + aadA*		6–64	4–48	1.5–2		3	1		1	
*aac(6′)-Iaa + aac(3)-IV + aph(4)-Ia + aadA*		8–12	16–24	1.5–3					2	
*aac(6′)-Iaa + aac(3)-IV + aph(4)-Ia + aph(3′)-Ia + aadA*		4–8	12–24	1.5–3		1			5	
*aac(6′)-Iaa + aac(3)-IV + aac3IId + aph(4)-Ia + aph(3′)-Ia + aph(6)-Id + aadA*		48	32	1.5					1	
*aac(6′)-Iaa + aac(3)-IV + aph(4)-Ia + aph(3″)-Ib + aph(6)-Id + aac(6′)-lb-cr*		12–16	48–256	4				3		
*aac(6′)-Iaa + aac(3)-IV + aph(4)-Ia + aadA + aac(6′)-lb-cr*		16	64–256	3–4			1	1		
*aac(6′)-Iaa + aac(3)-IV + aph(4)-Ia + aph(3′)-Ia + aadA + aac(6′)-lb-cr*		3.0	8	3		1				
*aac(6′)-Iaa + aac(3)-IV + aph(4)-Ia + aph(3″)-Ib + aph(6)-Id + aac(6′)-lb-cr + rmtB*		>256	>256	256				1		
		**CIP (R > 0.06)**		**NAL (ECOFF 8.0)**						
No genes		0.03		8			1			
*aac(6′)-Ib-cr*		0.06		6		1				
*parC*(T57S) + *qnrA1*		0.02		6						1
*qnrS1*		0.13–1.5		**8**–256		7	6		2	1
*parC*(T57S) + *qnrS1*		0.09–0.75		**8**–96		1	8		1	2
*qnrS1* + *aac(6′)-Ib-cr*		1.5		48			1			
*gyrA*(D87Y)		0.13		>256		1				
*gyrA*(D87Y) + *parC*(T57S)		0.19–0.25		>256		1			6	
*gyrA*(S83Y) + *parC*(T57S) + *qnrS1*		0.38–0.5		>256			2			
*gyrA*(D87Y) + *parC*(T57S) + *qnrS1*		0.13		>256					1	
*gyrA*(S83F) + *gyrA*(D87N) + *parC*(T57S) + *parC*(S80I)		>32		>256					1	
*gyrA*(S83F) + *gyrA*(D87G) + *parC*(T57S) + *parC*(S80R) + *aac(6′)-Ib-cr*		>32		>256				4		
*gyrA*(S83F) + *gyrA*(D87G) + *parC*(T57S) + *parC*(S80R) + *qnrS1* + *aac(6′)-Ib-cr*		>32		>256				1		
		**CHL (ECOFF 16.0)**								
No genes		2–16				1	2		1	1
*cmlA1*		64–192					4			
*cmlA1 + catB3*		192					1			
*floR*		>256				4	5		9	1
*floR + catA2*		>256				4				
*floR + catA3*		>256							1	
*floR + cmlA1*		>256				1	6			1
*floR + cmlA1 + catA2*		>256				1				
*floR + cmlA1 + catA3*		>256								1
*floR + cmlA1 + catB3*		>256						1		
*floR + catA1 + catB3*		>256						4		
		**TMP/SMX (R > 4)**								
No genes		0.05							1	
*sul1*		0.06–0.25							2	1
*sul2*		0.13–0.5				4	4			
*sul1* + *sul2*		0.19–0.25						4		
*sul1* + *sul2* + *sul3*		0.09					1			
*dfrA12* + *sul2*		>32					3			
*dfrA12* + *sul3*		>32					2			
*dfrA12* + *sul2* + *sul3*		>32				1	5			2
*dfrA12 + sul1* + *sul2* + *sul3*		>32				1		1		
*dfrA14 + sul1*		>32				1			5	
*dfrA14 + sul2*		>32				4				
*dfrA14 + sul1* + *sul2*		>32							1	
*dfrA14 +* + *sul3*		>32					1		2	1
*dfrA12 + dfrA5 + sul1* + *sul2* + *sul3*		>32					2			
		**AZM (ECOFF 16.0)**								
No genes		4–16				6	15	5	8	4
*mph(A)*		64–128				4	3		3	
*mef(B)*		32				1				
		**TET (ECOFF 8.0)**								
No genes		1–4				0	1	1		
*tetM*		4–6					2			
*tetA*		>256				4	2	3	10	3
*tetB*		>256				2	3			
*tetA* + *tetB*		>256				5	2		1	
*tetA* + *tetM*		>256					8	1		1
		**CT (R>2)**								
No genes		1–2				8	6	5	11	3
No genes		4–8				1	3			
*mcr* 1.1		8–16					6			1
*mcr* 3.1		4–8				2	2			
*mcr* 3.5		8					1			
MIC	Susceptible isolates									
**MIC**	Susceptible isolates (according clinical breakpoint or wild–type)									
MIC *	Non–wild type isolate. CTX ECOFF 0.5 mg/L									
MIC	Susceptible isolates. increased exposure									
MIC	Resistant isolates									

## Data Availability

All data of this study are presented in the article. The assembled genome sequences for all isolates were uploaded to the NCBI Sequence Read Archive under the project number PRJNA933104 at https://www.ncbi.nlm.nih.gov/bioproject/PRJNA933104 (accessed on 1 March 2024).

## Abbreviations

The following abbreviations are used in this manuscript:

NTS	Non-typhoidal Salmonella
WGS	Whole-genome sequencing
MDR	Multidrug resistance
ARG	Antimicrobial resistance genes
AST	Antimicrobial susceptibility testing
WT	Wild-type
NWT	Non-wild-type
BLs	Beta-lactamases
AMP	Ampicillin
CTZ	Ceftazidime
CTX	Cefotaxime
FEP	Cefepime
MEM	Meropenem
NAL	Nalidixic acid
CIP	Ciprofloxacin
TET	Tetracycline
CHL	Chloramphenicol
TMP/SMX	Trimethoprim/Sulfamethoxazole
CT	Colistin
GEN	Gentamycin
TOB	Tobramycin
AMI	Amikacin
AZM	Azithromycin

## References

[1] Nhung N.T., Phu D.H., Carrique-Mas J.J., Padungtod P. (2024). A review and meta-analysis of non-typhoidal Salmonella in Vietnam: Challenges to the control and antimicrobial resistance traits of a neglected zoonotic pathogen. One Health.

[2] Kim S., Kang H., Excler J.-L., Kim J.H., Lee J.-S. (2024). The Economic Burden of Non-Typhoidal Salmonella and Invasive Non-Typhoidal Salmonella Infection: A Systematic Literature Review. Vaccines.

[3] Billah M.M., Rahman M.S. (2024). Salmonella in the environment: A review on ecology, antimicrobial resistance, seafood contaminations, and human health implications. J. Hazard. Mater. Adv..

[4] Caneschi A., Bardhi A., Barbarossa A., Zaghini A. (2023). The Use of Antibiotics and Antimicrobial Resistance in Veterinary Medicine, a Complex Phenomenon: A Narrative Review. Antibiotics.

[5] Acosta A., Tirkaso W., Nicolli F., Van Boeckel T.P., Cinardi G., Song J. (2025). The future of antibiotic use in livestock. NaNat. Commun..

[6] Founou L.L., Founou R.C., Essack S.Y. (2016). Antibiotic Resistance in the Food Chain: A Developing Country-Perspective. Front. Microbiol..

[7] Sima C.M., Buzilă E.R., Trofin F., Păduraru D., Luncă C., Duhaniuc A., Dorneanu O.S., Nastase E.V. (2024). Emerging Strategies against Non-Typhoidal Salmonella: From Pathogenesis to Treatment. Curr. Issues Mol. Biol..

[8] Kumar G., Kumar S., Jangid H., Dutta J., Shidiki A. (2025). The rise of non-typhoidal Salmonella: An emerging global public health concern. Front. Microbiol..

[9] Hengkrawit K., Tangjade C. (2022). Prevalence and Trends in Antimicrobial Susceptibility Patterns of Multi-Drug-Resistance Non-Typhoidal Salmonella in Central Thailand, 2012–2019. Infect. Drug Resist..

[10] Sahu A.A., Sephalika S., Mohakud N.K., Sahu B.R. (2025). Prevalence and Multidrug Resistance in Non-Typhoidal Salmonella in India: A 20-Year Outlook. Acta Microbiol. Hell..

[11] Tuat C.V., Hue P.T., Loan N.T.P., Thuy N.T., Hue L.T., Giang V.N., Erickson V.I., Padungtod P. (2021). Antimicrobial Resistance Pilot Surveillance of Pigs and Chickens in Vietnam, 2017–2019. Front. Vet. Sci..

[12] Bloomfield S., Duong V.T., Tuyen H.T., Campbell J.I., Thomson N.R., Parkhill J., Le Phuc H., Chau T.T.H., Maskell D.J., Perron G.G. (2022). Mobility of antimicrobial resistance across serovars and disease presentations in non-typhoidal Salmonella from animals and humans in Vietnam. Microb. Genom..

[13] Holohan N., Wallat M., Luu T.H.Y., Clark E., Truong D.T.Q., Xuan S.D., Vu H.T.K., Van Truong D., Huy H.T., Nguyen-Viet H. (2022). Analysis of Antimicrobial Resistance in Non-typhoidal Salmonella Collected From Pork Retail Outlets and Slaughterhouses in Vietnam Using Whole Genome Sequencing. Front. Vet. Sci..

[14] Coyne L., Arief R., Benigno C., Giang V.N., Huong L.Q., Jeamsripong S., Kalpravidh W., McGrane J., Padungtod P., Patrick I. (2019). Characterizing Antimicrobial Use in the Livestock Sector in Three South East Asian Countries (Indonesia, Thailand, and Vietnam). Antibiotics.

[15] Redondo-Salvo S., Fernández-López R., Ruiz R., Vielva L., DE Toro M., Rocha E.P.C., Garcillán-Barcia M.P., De La Cruz F. (2020). Pathways for horizontal gene transfer in bacteria revealed by a global map of their plasmids. Nat. Commun..

[16] Commichaux S., Rand H., Javkar K., Molloy E.K., Pettengill J.B., Pightling A., Hoffmann M., Pop M., Jayeola V., Foley S. (2023). Assessment of plasmids for relating the 2020 Salmonella enterica serovar Newport onion outbreak to farms implicated by the outbreak investigation. BMC Genom..

[17] Yan S., Jiang Z., Zhang W., Liu Z., Dong X., Li D., Liu Z., Li C., Liu X., Zhu L. (2023). Genomes-based MLST, cgMLST, wgMLST and SNP analysis of Salmonella Typhimurium from animals and humans. Comp. Immunol. Microbiol. Infect. Dis..

[18] Mather A.E., Phuong T.L.T., Gao Y., Clare S., Mukhopadhyay S., Goulding D.A., Hoang N.T.D., Tuyen H.T., Lan N.P.H., Thompson C.N. (2018). New Variant of Multidrug-Resistant Salmonella enterica Serovar Typhimurium Associated with Invasive Disease in Immunocompromised Patients in Vietnam. mBio.

[19] Soliani L., Rugna G., Prosperi A., Chiapponi C., Luppi A. (2023). Salmonella Infection in Pigs: Disease, Prevalence, and a Link between Swine and Human Health. Pathogens.

[20] Tu L.T.P., Hoang N.V.M., Cuong N.V., Campbell J., Bryant J.E., Hoa N.T., Kiet B.T., Thompson C., Duy D.T., Phat V.V. (2015). High levels of contamination and antimicrobial-resistant non-typhoidal Salmonella serovars on pig and poultry farms in the Mekong Delta of Vietnam. Epidemiol. Infect..

[21] Nguyen D.T.A., Kanki M., Nguyen P.D., Le H.T., Ngo P.T., Tran D.N.M., Le N.H., Van Dang C., Kawai T., Kawahara R. (2016). Prevalence, antibiotic resistance, and extended-spectrum and AmpC beta-lactamase productivity of Salmonella isolates from raw meat and seafood samples in Ho Chi Minh City, Vietnam. Int. J. Food Microbiol..

[22] Ta Y.T., Nguyen T.T., To P.B., Pham D.X., Le H.T.H., Thi G.N., Alali W.Q., Walls I., Doyle M.P. (2014). Quantification, serovars, and antibiotic resistance of salmonella isolated from retail raw chicken meat in Vietnam. J. Food Prot..

[23] Leinyuy J.F., Ali I.M., Ousenu K., Tume C.B. (2023). Molecular characterization of antimicrobial resistance related genes in E. coli, Salmonella and Klebsiella isolates from broilers in the West Region of Cameroon. PLoS ONE.

[24] Nhung N.T., Van N.T.B., Van Cuong N., Duong T.T.Q., Nhat T.T., Hang T.T.T., Nhi N.T.H., Kiet B.T., Hien V.B., Ngoc P.T. (2017). Antimicrobial residues and resistance against critically important antimicrobials in non-typhoidal Salmonella from meat sold at wet markets and supermarkets in Vietnam. Int. J. Food Microbiol..

[25] Da Pham X., Hong H.L.T., Thanh H.T.T., Le L.T., Le H.V., Thi N.H., Le Tran M., Trung N.T. (2023). Strains and virulence genes of salmonella with multidrug resistance isolated from chicken carcasses (Ha-noi, Vietnam). Health Risk Analysis.

[26] Nguyen T.T., Le H.V., Hai H.V.T., Tuan T.N., Nguyen H.M., Xuan D.P., Thanh H.T.T., Le Thi H.H. (2023). Whole-Genome Analysis of Antimicrobial-Resistant Salmonella enterica Isolated from Duck Carcasses in Hanoi, Vietnam. Curr. Issues Mol. Biol..

[27] Bush K., Jacoby G.A. (2010). Updated functional classification of beta-lactamases. Antimicrob. Agents Chemother..

[28] Song Q., Xu Z., Gao H., Zhang D. (2018). Overview of the development of quinolone resistance in Salmonella species in China, 2005-2016. Infect. Drug Resist..

[29] Pribul B.R., Festivo M.L., Rodrigues M.S., Costa R.G., Rodrigues E.C.d.P., de Souza M.M.S., Rodrigues D.d.P. (2017). Characteristics of Quinolone Resistance in Salmonella spp. Isolates from the Food Chain in Brazil. Front. Microbiol..

[30] Weng R., Gu Y., Zhang W., Hou X., Wang H., Tao J., Deng M., Zhou M., Zhao Y. (2022). Corrigendum: Whole-genome sequencing provides insight into antimicrobial resistance and molecular characteristics of Salmonella from livestock meat and diarrhea patient in Hanzhong, China. Front. Microbiol..

[31] Kotb D.N., Mahdy W.K., Mahmoud M.S., Khairy R.M.M. (2019). Impact of co-existence of PMQR genes and QRDR mutations on fluoroquinolones resistance in Enterobacteriaceae strains isolated from community and hospital acquired UTIs. BMC Infect. Dis..

[32] Navickaite I., Holmes H., Dondi L., Randall L., Fearnley C., Taylor E., Fullick E., Horton R., Williamson S., AbuOun M. (2024). Occurrence and characterization of rmtB-harbouring Salmonella and Escherichia coli isolates from a pig farm in the UK. J. Antimicrob. Chemother..

[33] Wang J., Wang Z.-Y., Wang Y., Sun F., Li W., Wu H., Shen P.-C., Pan Z.-M., Jiao X. (2021). Emergence of 16S rRNA Methylase Gene rmtB in Salmonella Enterica Serovar London and Evolution of RmtB-Producing Plasmid Mediated by IS26. Front. Microbiol..

[34] Zhang Y., Chen J., Yang X., Wu Y., Wang Z., Xu Y., Zhou L., Wang J., Jiao X., Sun L. (2025). Emerging Mobile Colistin Resistance Gene Mcr-1 and Mcr-10 in Enterobacteriaceae Isolates From Urban Sewage in China. Infect. Drug Resist..

[35] Fortini D., Owczarek S., Dionisi A.M., Lucarelli C., Arena S., Carattoli A., Villa L., García-Fernández A., Enter-Net Italia Colistin Resistance Study Group (2022). Colistin Resistance Mechanisms in Human Salmonella enterica Strains Isolated by the National Surveillance Enter-Net Italia (2016–2018). Antibiotics.

[36] Pavelquesi S.L.S., Ferreira A.C.A.d.O., Rodrigues A.R.M., Silva C.M.d.S., Orsi D.C., da Silva I.C.R. (2021). Presence of Tetracycline and Sulfonamide Resistance Genes in Salmonella spp.: Literature Review. Antibiotics.

[37] Bryan A., Shapir N., Sadowsky M.J. (2004). Frequency and distribution of tetracycline resistance genes in genetically diverse, nonselected, and nonclinical Escherichia coli strains isolated from diverse human and animal sources. Appl. Environ. Microbiol..

[38] Jurado-Rabadán S., de la Fuente R., A Ruiz-Santa-Quiteria J., Orden J.A., de Vries L.E., Agersø Y. (2014). Detection and linkage to mobile genetic elements of tetracycline resistance gene tet(M) in Escherichia coli isolates from pigs. BMC Vet. Res..

[39] Chia-Wei L., Cheng J.-F., Tung K.-C., Hong Y.-K., Lin J.-H., Lin Y.-H., Tsai C.-A., Lin S.-P., Chen Y.-C., Shi Z.-Y. (2022). Evolution of trimethoprim/sulfamethoxazole resistance in Shewanella algae from the perspective of comparative genomics and global phylogenic analysis. J. Microbiol. Immunol. Infect..

[40] (2017). Microbiology of the Food Chain—Horizontal Method for the Detection, Enumeration and Serotyping of Salmonella—Part 1: *Detection of salmonella* spp..

